# Is self-reported depression, HIV status, COVID-19 health risk profile and SARS-CoV-2 exposure associated with difficulty in adhering to COVID-19 prevention measures among residents in West Africa?

**DOI:** 10.1186/s12889-022-14429-6

**Published:** 2022-11-10

**Authors:** Morenike Oluwatoyin Folayan, Roberto Ariel Abeldaño Zuniga, Giuliana Florencia Abeldaño, Mir Faeq Ali Quadri, Mohammed Jafer, Muhammad Abrar Yousaf, Passent Ellakany, Ntombifuthi Nzimande, Eshrat Ara, Nuraldeen Maher Al-Khanati, Zumama Khalid, Folake Lawal, Mark Roque, Joanne Lusher, Bamidele O. Popoola, Abeedha Tu-Allah Khan, Martin Amogre Ayanore, Balgis Gaffar, Jorma I. Virtanen, Nourhan M. Aly, Joseph Chukwudi Okeibunor, Maha El Tantawi, Annie Lu Nguyen

**Affiliations:** 1 Mental Health and Wellness Study Group, Obafemi Awolowo University, Ile-Ife, Nigeria; 2 Department of Child Dental Health, Obafemi Awolowo University, Ile-Ife, Nigeria; 3 Nigeria Institute of Medical Research, Lagos, Nigeria; 4 Postgraduate Department, University of Sierra Sur., Oaxaca, Mexico; 5 School of Medicine, University of Sierra Sur., Oaxaca, Mexico; 6 Department of Oral Health Sciences, School of Dentistry, University of Washington, Seattle, USA; 7 Division of Dental Public Health, Jazan University, Jazan, Saudi Arabia; 8 Department of Health Promotion, Faculty of Health, Medicine and Life Sciences, Maastricht University, Maastricht, The Netherlands; 9 Department of Biology, Virtual University of Pakistan, Lahore, Pakistan; 10 Department of Substitutive Dental Sciences, College of Dentistry, Imam Abdulrahman Bin Faisal University, Dammam, Saudi Arabia; 11 Department of Economic and Human Geography, Faculty of Geosciences, University of Szeged, 6722 Szeged, Hungary; 12 Department of Psychology, Government College for Women, MA Road Srinagar Kashmir, Jammu and Kashmir, India; 13 Department of Oral and Maxillofacial Surgery, Faculty of Dentistry, Syrian Private University, Damascus, Syria; 14 Department of Health Sciences, University of Genova, 16132 Genoa, GE Italy; 15 Department of Periodontology and Community Dentistry, University of Ibadan and University College Hospital, Ibadan, Nigeria; 16 Maternity and Childhood Nursing Department, College of Nursing, Taibah University, Madinah, Kingdom of Saudi Arabia; 17 Regent’s University London, London, UK; 18 Department of Child Oral Health, University of Ibadan, Ibadan, Nigeria; 19 School of Biological Sciences, University of the Punjab, Quaid-E-Azam Campus, Lahore, 54590 Pakistan; 20 Department of Health Policy Planning and Management, University of Health and Allied Sciences, Ho, Ghana; 21 Department of Preventive Dental Sciences, College of Dentistry, Imam Abdulrahman Bin Faisal University, Dammam, Saudi Arabia; 22 Faculty of Medicine, University of Turku, Turku, Finland; 23 Department of Pediatric Dentistry and Dental Public Health, Faculty of Dentistry, Alexandria University, Alexandria, Egypt; 24 World Health Organisation, AFRO, Addis Ababa, Ethiopia; 25 Department of Family Medicine, Keck School of Medicine, University of Southern California, Los Angeles, USA

**Keywords:** Risk compensation, COVID-19 preventive measures, SARS-CoV-2, AIDS, Mental Wellness, Mental health, HIV, West Africa

## Abstract

**Background:**

The aim of this study was to determine whether self-reported depression, coronavirus disease of 2019 (COVID-19) health risk profile, HIV status, and SARS-CoV-2 exposure were associated with the use of COVID-19 prevention measures.

**Methods:**

This survey collected data electronically between June 29 and December 31, 2020 from a convenient sample of 5050 adults 18 years and above living in 12 West African countries. The dependent variables were: social distancing, working remotely, difficulty obtaining face masks and difficulty washing hands often. The independent variables were self-reported depression, having a health risk for COVID-19 (high, moderate and little/no risk), living with HIV and COVID-19 status (SARS-CoV-2 positive tests, having COVID-19 symptoms but not getting tested, having a close friend who tested positive for SARS-CoV-2 and knowing someone who died from COVID-19). Four binary logistic regression models were developed to model the associations between the dependent and independent variables, adjusting for socio-demographic variables (age, gender, educational status, employment status and living status).

**Results:**

There were 2412 (47.8%) male participants and the mean (standard deviation) age was 36.94 (11.47) years. Respondents who reported depression had higher odds of working remotely (AOR: 1.341), and having difficulty obtaining face masks (AOR: 1.923;) and washing hands often (AOR: 1.263). People living with HIV had significantly lower odds of having difficulty washing hands often (AOR: 0.483). Respondents with moderate health risk for COVID-19 had significantly higher odds of social distancing (AOR: 1.144) and those with high health risk had difficulty obtaining face masks (AOR: 1.910). Respondents who had a close friend who tested positive for SARS-CoV-2 (AOR: 1.132) and knew someone who died of COVID-19 (AOR: 1.094) had significantly higher odds of social distancing. Those who tested positive for SARS-CoV-2 had significantly lower odds of social distancing (AOR: 0.629) and working remotely (AOR: 0.713). Those who had symptoms of COVID-19 but did not get tested had significantly lower odds of social distancing (AOR: 0.783) but significantly higher odds of working remotely (AOR: 1.277).

**Conclusions:**

The study signifies a disparity in the access to and use of COVID-19 preventative measures that is allied to the health and COVID-19 status of residents in West Africa. Present findings point to risk compensation behaviours in explaining this outcome.

**Supplementary Information:**

The online version contains supplementary material available at 10.1186/s12889-022-14429-6.

## Introduction

West Africa is a region with the second lowest cases of coronavirus disease of 2019 (COVID-19) in Africa behind Central Africa [1, 2]. The reported COVID-19 cases are over 825,800 with approximately 11,200 deaths as at 6^th^ of February 2022 [3]. There is a serious concern over the inadequate coverage of COVID-19 vaccination in the West African region – with only 0.27% of the total population receiving the complete vaccine doses three months after launching vaccination campaigns. Furthermore, it is estimated that less than 1.6% of the region's total population will be fully vaccinated 18 months after the vaccine deployment [4]. The adoption and use of COVID-19 prevention strategies will continue to be the mainstay for infection control in West Africa for a long time to come while efforts are being made to increase vaccine access [5]. The region is home to 16 countries all classified as low and low middle-income countries, with an estimated population of over 401 million people in 2020 accounting for about 5% of the world’s population [6]. As a resource-constrained region, adopting low-cost but effective strategies to control the spread of COVID-19 is essential. The combination of quarantine, contact tracing, screening, and isolation are effective COVID-19 prevention measures when integrated [7]. However, COVID-19 surveillance and contact tracing had been poorly implemented in West Africa, increasing the risk for continued COVID-19 transmission [8]. Five countries in the region—Benin, Burkina Faso, Mali, Mauritania, Nigeria—are currently experiencing the fourth wave of the pandemic [9]. In addition to getting vaccinated, COVID-19 preventive measures include keeping social distance and working from home to reduce close contact with others, wearing a face mask that covers the nose and mouth in public settings and regularly washing hands with soap and water for at least 20 s, or using an alcohol-based hand sanitiser that contains at least 60% alcohol [10]. Social distancing was demonstrated to have prevented about 84% of COVID-19 cases and about 66% of COVID-19 related deaths compared with what would have occurred without a social distancing policy within three weeks of the first wave of the epidemic in Germany [11]. Social distancing reduced the number of new cases, hospitalizations, and deaths and provided time to help healthcare systems cope with the crisis. It is most effective when done in conjunction with testing and contact tracing [12]. It is, most effective when done in conjunction with testing and contact tracing [12]. A few countries have relaxed their policy on active contact tracking for COVID-19 prevention [13–18]; and many countries in sub-Saharan Africa have done poorly with contact tracing [19–21]. Understanding factors that can otherwise, reduce the use of COVID-19 preventive measures in West Africa is, therefore, essential.

According to one study, community face mask wearing is also an effective intervention to reduce the spread of COVID-19 by limiting the exhalation and inhalation of the causative virus [22]. Significant public health benefits are achieved only when compliance is high [23]. Handwashing is also an important COVID-19 prevention strategy because hands are a vector for cross-transmission of microorganisms [24] and viral infections [25] including severe acute respiratory syndrome coronavirus 2 (SARS-CoV-2) [26].

The implementation of COVID-19 prevention measures, including lockdowns, resulted in economic losses for many individuals. High rates of unemployment with associated social and psychological costs [27, 28], closure of schools, retail markets, event centres, places of worship, and separation from family members increased the risk of depression for citizens in the region [29]. Also, concerns about the risk of death from COVID-19, misinformation, disinformation, poor access to vaccination and health care in addition to stigma and discrimination associated with COVID-19 [30] exacerbated the risk of COVID-19 related mental problems. The prevalence of depression is as high as 30% in sub-Saharan Africa [31] Prior studies suggest that people with depression and mental challenges complied better with preventive measures [32, 33]. Another study however, reported that depression was associated with lower compliance with COVID-19 precautionary measures [34]. In West Africa, the risk of depression before COVID-19 was high due to exposure to adverse events like war, sexual abuse, violence, diseases, pestilence, and emotional neglect [35]. The additional trauma from COVID-19 is likely to increase the prevalence of depression; and it is important to understand how depression may affect compliance with key COVID-19 containing measures in the region.

HIV is also endemic in the region with about 5 million people living with the infection in West Africa [36]. There are concerns about people living with HIV being at higher risk for COVID-19, and COVID-19 related deaths. It is important to understand the level of compliance of people living with HIV in the region to COVID-19 prevention measures.

The study aimed to determine the associations between self-reported experience of depression, HIV status, COVID-19 health risk profile, COVID-19 status on one hand, and the use of COVID-19 prevention measures (social distancing, working remotely, using face mask and hand washing/sanitising) on the other hand. We postulated that self-reported depression, HIV status, COVID-19 health risk profile, COVID-19 status will be positively associated with the use of COVID-19 prevention measures.

## Methods

### Ethical approval and consent to participate

Ethical approval for the study was obtained from the Human Research Ethics Committee at the Institute of Public Health of the Obafemi Awolowo University Ile-Ife, Nigeria (HREC No: IPHOAU/12/1557). Data were collected anonymously, and the confidentiality, privacy, rights, and welfare of the research participants were safeguarded through measures taken to prevent the unintended collection of IP addresses. Informed consent was obtained from the study participants for the online survey by asking them to tick a checkbox that indicated consenting to join the study. Participants could only proceed to the survey after ticking the checkbox. The study was performed in accordance with the National Health Research Ethics Code of Nigeria [37].

### Study design, study participants and study participants’ recruitment

We extracted data of respondents’ living in any of the 16 countries in West Africa during the COVID-19 pandemic from a dataset of a global survey on the impact of the COVID-19 on the mental health and wellness of adults. This survey collected data from adults ages 18 years and above between June 29^th^ and December 31^st^ 2020 during the first wave of the COVID-19 pandemic. There were no other exclusion criteria. The study participants were recruited through respondent-driven convenient sampling. The 45 data collectors who are members of the Mental Health and Wellness Research Group [38] shared the survey link with their contacts to facilitate recruitment (snowball sampling). The survey link was also posted on social media platforms (Facebook, Twitter, Instagram, WhatsApp). More details on the study methodology can be found in prior publications [39, 40].

### Study instruments

The questionnaire was preceded by a brief introduction explaining the purpose of the study, and assuring respondents of their voluntary participation, and the confidentiality of their data. The questionnaire took about 11 min to complete. Participants could fill out the English, French, Portuguese, Spanish, or Arabic versions of the questionnaire. Each participant could only complete a single questionnaire through IP address restrictions though they could edit their answers freely until they chose to submit. The content validity index for the questionnaire was 0.82 [41].

### Dependent variables: use of COVID-19 prevention strategies

The questionnaire measured the use of COVID-19 prevention strategies: maintaining social distancing, difficulty for obtaining a face mask, difficulty with hands washing as often as recommended, and working remotely. Respondents were asked whether they adopted the listed behaviour during the pandemic. Respondents could select more than one item if they adopted multiple behaviours during the pandemic. The questions were included as a component of the Pandemic Stress Index [41].

### Independent variables

Depression, health profile, HIV status [39, 42], and exposure to SARS-CoV-2 [43] were factors included in the study model to test their association with the use of COVID-19 prevention strategies. Respondents reported their experience of depression by ticking a checklist of eight feelings that they may have experienced during the COVID-19 pandemic. Respondents who ticked depression were identified as having felt depressed during the pandemic. The variable was dichotomous (yes/no). This item was part of the Pandemic Stress Index assessing the psychosocial impact of COVID-19 [41].

Respondents were asked if they had any of 23 health conditions on a checklist in addition to other health conditions not listed. The health conditions were categorised into those putting individuals at high risk for COVID-19 (pneumonia, asthma, diabetes, cancer, heart condition), those putting people at moderate risk of COVID-19 (hepatitis, respiratory problems, hypertension, stroke, neurological problems, neuropathy) and those placing people at little or no risk for COVID-19 (dermatologic problems, migraines, arthritis, broken bones, hearing loss and vision loss, herpes, shingles and other sexually transmitted infections). Respondents were also able to free list other health problems which were then categorised into a risk profile after review of the literature [44]. As part of the 23 listed health conditions, participants were asked about their HIV status and they could report if they were living with HIV by ticking a checkbox. All respondents who did not tick this checkbox were categorised as not living with HIV. Respondents were also asked if they had ever tested positive to SARS-CoV-2, had COVID-19 symptoms but did not get tested, had a close friend who tested positive for SARS-CoV-2, or knew someone who died from COVID-19. Choices for these items were ‘yes’ or ‘no’ [44].

### Confounders

Factors that may be associated with the use of COVID-19 prevention strategies and depression include the socio-demographic profile – depression is more prevalent in older people due to worsening medical conditions [45], and younger persons are less likely to adhere to COVID-19 prevention strategies [46]. Other potential confounders of the study are gender [47, 48], educational status [49, 50] and employment status [51, 52].

Data were collected on the age of respondents at last birthday, sex at birth (male, female, intersex, decline to answer), the highest level of education attained (no formal education, primary, secondary, college/university), employment status (job loss – yes/no; and loss of wages – yes/no), and living status (living with others – yes/no). Sex at birth was dichotomised into males and non-males.

### Statistical analysis

Raw data were downloaded as SPSS® file Version 23.0 (IBM Corp. Armonk, NY, USA). T-test and chi square test were used to assess the relationship between the dependent variable, independent variables, and confounders. Four binary logistic regression models were constructed to identify whether depression, medical health profile, HIV status, exposure to SARS-CoV-2 infection were associated with the use of the four COVID-19 precautionary measures after adjusting for potential confounders. Adjusted odds ratios (AOR) and 95% confidence intervals (CIs) were calculated. Statistical significance was set at 5%.

## Results

Table 1 showed the profile of the 5050 study participants recruited from 12 countries in West Africa (Supplemental File 1). The mean (standard deviation) age of the study participants was 36.94 (11.47) years. Of the 5050 respondents, 307 (6.1%) reported feeling depressed, 181 (3.6%) had a high COVID-19 health risk profile, 941 (18.6%) were living with HIV, and 133 (2.6%) reported a history of COVID-19 positive status. Further, 3571 (70.7%) practised social distancing, 4749 (94.0%) wore a face mask, 4856 (96.2%) washed their hands often, and 1480 (29.3%) worked remotely.

**Table 1:** Factors associated with adopting COVID-19 prevention measures (social distancing, difficulty obtaining a face mask, difficulty washing hands often and working remotely) by adults in West Africa (*N* = 5050)

Variables	Total *N* = 5050	Social distancing			Difficulty obtaining a mask or face covering			Difficulty washing hands often			Work remotely		
		**No = 1479n (%)**	**Yes = 3571n (%)**	***p* value**	**No = 4749n (%)**	**Yes = 301n (%)**	***p* value**	**No = 4856n (%)**	**Yes = 194n (%)**	***p* value**	**No = 3570n (%)**	**Yes = 1480n (%)**	***p* value**
**Age**		Mean 35.1 (11.5)	Mean 37.7 (11.4)	**< 0.001**	Mean 37.1 (11.6)	Mean 35.1 (8.8)	**0.001**	Mean 37.0 (11.5)	Mean 34.2 (10.8)	**0.001**	Mean 36.0 (11.5)	Mean 39.0 (11.0)	**< 0.001**
**Gender**													
Males	2412	674 (45.6)	1738 (48.7)	**0.045**	2253 (47.4)	159 (52.8)	0.070	2308 (47.5)	104 (53.6)	0.096	1666 (46.7)	746 (50.4)	**0.015**
Non-males	2638	805 (54.4)	1833 (51.3)		2496 (52.6)	142 (47.2)		2548 (52.5)	90 (46.4)		1904 (53.3)	734 (49.6)	
**Level of education**													
No formal education	53	38 (2.6)	15 (0.4)	**< 0.001**	42 (0.9)	11 (3.7)	**< 0.001**	50 (1.0)	3 (1.5)	**0.014**	48 (1.3)	5 (0.3)	**< 0.001**
Primary	88	50 (3.4)	38 (11.1)		83 (1.7)	5 (1.7)		84 (1.7)	4 (2.1)		81 (2.3)	7 (0.5)	
Secondary	787	317 (21.4)	470 (13.2)		752 (15.8)	35 (11.6)		755 (15.5)	32 (16.5)		683 (19.1)	104 (7.0)	
Tertiary	4122	1074 (72.6)	3048 (85.4)		3872 (81.5)	250 (83.1)		3967 (81.7)	155 (79.9)		2758 (77.3)	1364 (92.2)	
**Job los**													
No	4668	1386 (93.7)	3282 (91.9)	**0.027**	4388 (92.4)	280 (93.0)	0.691	4492 (92.5)	176 (90.7)	0.357	3261 (91.3)	1407 (95.1)	**< 0.001**
Yes	382	93 (6.3)	289 (8.1)		361 (7.6)	21 (7.0)		364 (7.5)	18 (9.3)		309 (8.7)	73 (4.9)	
**Reduced wages**													
No	3771	1175 (79.4)	2596 (72.7)	**< 0.001**	3542 (74.6)	229 (76.1)	0.563	3637 (74.9)	134 (69.1)	0.067	2769 (77.6)	1002 (67.7)	**< 0.001**
Yes	1279	304 (20.6)	975 (27.3)		1207 (25.4)	72 (23.9)		1219 (25.1)	60 (30.9)		801 (22.4)	478 (32.3)	
**Living with others**													
No	1089	348 (23.5)	741 (20.8	**0.029**	1015 (21.4	74 (24.6	0.189	1044 (21.5	45 (23.2	0.573	788 (22.1	301 (20.3)	0.172
Yes	3961	1131 (76.5)	2830 (79.2)		3734 (78.6)	227 (75.4)		3812 (78.5)	149 (76.8)		2782 (77.9)	1179 (79.7)	
**COVID-19 health risk profile**													
High risk	186	56 (3.8)	130 (3.6)	0.553	171 (3.6)	15 (5.0)	**0.005**	174 (3.6)	12 (6.2)	0.062	121 (3.4)	65 (4.4)	0.096
Moderate risk	679	187 (12.6)	492 (13.8)		622 (13.1)	57 (18.9)		647 (13.3)	32 (16.5)		467 (13.1)	212 (14.3)	
Little or no risk	4185	1236 (83.6)	2949 (82.6)		3956 (83.3)	229 (76.1)		4035 (83.1)	150 (77.3)		2982 (83.5)	1203 (81.3)	
**Exposure to SARS-CoV-2 infection**													
Tested positive for SARS-CoV-2													
No	4917	1438 (97.2)	3479 (97.4)	0.692	4626 (97.4)	291 (96.7)	0.442	4728 (97.4)	189 (97.4)	0.960	3475 (97.3)	1442 (97.4)	0.850
Yes	133	41 (2.8)	92 (2.6)		123 (2.6)	10 (3.3)		128 (2.6)	5 (2.6)		95 (2.7)	38 (2.6)	
Had COVID-19 symptoms but did not get tested													
No	4512	1305 (88.2)	3207 (89.8)	0.100	4258 (89.7)	254 (84.4)	**0.004**	4351 (89.6)	161 (83.0)	**0.003**	3182 (89.1)	1330 (89.9)	0.442
Yes	538	174 (11.8)	364 (10.2)		491 (10.3)	47 (15.6)		505 (10.4)	33 (17.0)		388 (10.9)	150 (10.1)	
Had a close friend who tested positive for SARS-CoV-2													
No	4192	1284 (86.8)	2908 (81.4)	**< 0.001**	3967 (83.5)	225 (74.8)	**< 0.001**	4039 (83.2)	153 (78.9)	0.117	2988 (83.7)	1204 (81.4)	**0.043**
Yes	858	195 (13.2)	663 (18.6)		782 (16.5)	76 (25.2)		817 (16.8)	41 (21.1)		582 (16.3)	276 (18.6)	
Knew someone who died from COVID-19													
No	3508	1130 (76.4)	2378 (66.6)	**< 0.001**	3320 (69.9)	188 (62.5)	**0.006**	3380 (69.6)	128 (66.0)	0.282	2555 (71.6)	953 (64.4)	**< 0.001**
Yes	1542	349 (23.6)	1193 (33.4)		1429 (30.1)	113 (37.5)		1476 (30.4)	66 (34.0)		1015 (28.4)	527 (35.6)	
**HIV status**													
Not living with HIV	4109	1119 (75.7)	2990 (83.7)	**< 0.001**	3846 (81.0)	263 (87.4)	**0.006**	3940 (81.1)	169 (87.1)	**0.036**	2762 (77.4)	1347 (91.0)	**0.001**
Living with HIV	941	360 (24.3)	581 (6.3)		903 (19.0)	38 (12.6)		916 (18.9)	25 (12.9)		808 (22.6)	133 (9.0)	
**Reported depression**													
No	4743	1358 (91.8)	3385 (94.8)	**< 0.001**	4474 (94.2)	269 (89.4)	**0.001**	4575 (94.2)	168 (86.6)	**< 0.001**	3341 (93.6)	1402 (94.7)	0.121
Yes	307	121 (8.2)	186 (5.2)		275 (5.8)	32 (10.6)		281 (5.8)	26 (13.4)		229 (6.4)	78 (5.3)	

Table 1 shows that there were significantly more non-depressed respondents who practiced social distancing during the COVID-19 pandemic (*p* < 0.001). Also, more respondents who were older (*p* < 0.001), non-males (*p* = 0.045), had primary and tertiary education (*p* < 0.001), had lost their jobs (*p* = 0.027), had reduced wages (*p* < 0.001), had a close friend who tested positive for SARS-CoV-2 (*p* < 0.001), knew someone who died from COVID-19 (*p* < 0.001), not living with HIV (*p* < 0.001), and living with others (*p *= 0.029) practiced social distancing. In addition, a significant number of respondents who were depressed had difficulty obtaining face masks (*p* = 0.001). Also, many respondents had difficulty in obtaining face masks. These respondents were younger (*p *= 0.001), had no formal and tertiary education (*p* < 0.001), who were at high risk of COVID (*p* = 0.005), had COVID-19 symptoms but did not get tested (*p* = 0.004), had a close friend who tested positive for SARS-CoV-2 (*p* < 0.001), knew someone who died from COVID-19 (*p* = 0.006) and not living with HIV (*p* = 0.006).

Further, there were a significant number of respondents who were depressed had difficulty in washing their hands often (*p* < 0.001). Also, a considerable number of respondents who were younger (*p *= 0.001), had no formal and primary education (*p* < 0.001), who were at high risk of COVID-19 (*p* = 0.005), had COVID-19 symptoms but did not get tested (*p* = 0.003), and not living with HIV (*p* = 0.036) had difficulty in washing their hands often.

Finally, respondents who were working remotely were: older (*p* < 0.001), males (*p* = 0.015), had tertiary education (*p* < 0.001), did not lose their job (*p* < 0.001), had reduced wages (*p* < 0.001), had a close friend who tested positive for SARS-CoV-2 (*p* = 0.043), knew someone who died from COVID-19 (*p* < 0.001) and not living with HIV (*p* = 0.001).

Table 2 shows that though reported depression and living with HIV were not significantly associated with social distancing, the health profile and exposure to SARS-CoV-2 infection were significantly associated with social distancing. Respondents at moderate risk of COVID-19 (AOR: 1.144); who had a close friend who tested positive for SARS-CoV-2 (AOR: 1.132); and who knew someone who died of COVID-19 (AOR: 1.094) had significantly higher odds of social distancing. Those who tested positive for SARS-CoV-2 (AOR: 0.629) or had symptoms of COVID-19 but did not get tested (AOR: 0.783) had significantly lower odds of social distancing.

**Table 2:** Logistic regression of factors associated with adopting COVID-19 prevention measures (social distancing, difficulty obtaining face mask, difficulty washing hands often and work remotely) by adults in West Africa (*N* = 5050)

Variables	Social distancing		Working remotely		Difficulty obtaining face mask		Difficulty washing hands often	
	AOR (95% C.I.)	*p *value	AOR (95% C.I.)	*p *value	AOR (95% C.I.)	*p *value	AOR (95% C.I.)	*p *value
**Age**	1.025 (1.022–1.028)	< 0.001	0.980 (0.975 – 0.985)	< 0.001	0.969 (0.962–0.977)	< 0.001	1.011 (1.009–1.014)	< 0.001
**Gender**								
None males (Ref: Males)	0.990 (0.925–1.059)	0.762	0.897 (0.797-`1.009)	0.070	0.783 (0.671–0.914)	0.002	1.052 (0.985–1.124)	0.132
**Educational status** (Ref: No formal education)								
Primary	1.731 (1.218–2.460)	0.002	0.595 (0.294–1.203)	0.149	1.290 (0.500–3.328)	0.598	0.846 (0.561–1.275)	0.424
Secondary	6.139 (4.589–8.212)	< 0.001	0.922 (0.559–1.523)	0.752	1.490 (0.686–3.238)	0.314	1.388 (1.014–1.901)	0.041
Tertiary	9.174 (6.901–12.196)	< 0.001	1.302 (0.802–2.114)	0.285	1.899 (0.889–4.058)	0.098	2.601 (1.918–3.529)	< 0.001
**Employment status**								
Job loss (Ref: No)	1.341 (1.192–1.508)	< 0.001	1.437 (1.205–1.715)	< 0.001	1.121 (0.876–1.436)	0.364	0.872 (0.776–0.980)	0.022
Reduced wages (Ref: No)	1.609 (1.476–1.755)	< 0.001	1.362 (1.192–1.557)	< 0.001	1.324 (1.108–1.582)	0.002	1.536 (1.421–1.659)	< 0.001
Living with others (Ref: Yes)	1.027 (0.936–1.126)	0.573	1.037 (0.884–1.217)	0.653	1.081 (0.877–1.333)	0.464	1.085 (0.993–1.187)	0.072
**COVID-19 health risk profile** (Ref: Little or no risk)								
Moderate risk	1.144 (1.003–1.304)	0.045	1.227 (0.984–1.529)	0.069	0.775 (0.554–1.085)	0.138	1.049 (0.931–1.182)	0.435
High risk	0.889 (0.760–1.039)	0.139	1.281 (0.977–1.680)	0.073	1.910 (1.387–2.632)	< 0.001	1.055 (0.910–1.224)	0.476
**Exposure to SARS-CoV-2 infection**								
I have tested positive for SARS-CoV-2 (Ref: No)	0.629 (0.542–0.731)	< 0.001	0.713 (0.536–0.948)	0.020	0.730 (0.501–1.063)	0.101	0.773 (0.659–0.907)	0.002
Had symptoms but did not get tested for SARS-CoV-2 (Ref: No)	0.783 (0.708–0.866)	< 0.001	1.277 (1.081–1.509)	0.004	1.184 (0.948–1.478)	0.136	0.901 (0.812–0.999)	0.049
Have a close friend who tested positive to SARS-CoV-2 (Ref: No)	1.132 (1.050–1.220)	0.001	1.077 (0.947–1.224)	0.260	1.056 (0.890–1253)	0.529	1.049 (0.976–1.127)	0.190
Know someone who died of COVID-19 (Ref: No)	1.094 (1.020–1.174)	0.012	1.024 (0.906–1.159)	0.701	1.033 (0.877–1.216)	0.699	1.036 (0.967–1.109)	0.313
**HIV status**								
Living with HIV (Ref: Not living with HIV)	0.871 (0.758–1.002)	0.053	0.981 (0.754–1.277)	0.886	1.041 (0.739–1.467)	0.817	0.483 (0.410–0.568)	< 0.001
**Depression** (Ref: No)	1.090 (0.929–1.278)	0.291	1.341 (1.044–1.722)	0.022	1.923 (1.348–2.744)	< 0.001	1.263 (1.090–1.464)	0.002

Respondents who reported being depressed (AOR: 1.341) and who had symptoms but did not get tested for COVID-19 (AOR: 1.277) had significantly higher odds of working remotely. Those who tested positive for SARS-CoV-2 (AOR: 0.713) had significantly lower odds of working remotely. HIV status was not associated with working remotely.

Respondents who reported being depressed (AOR: 1.923) and who had high risk of COVID-19 (AOR: 1.910) had significantly higher odds of difficulty obtaining face mask. HIV status was not associated with difficulty obtaining face mask.

Respondents who reported being depressed had significantly higher odds of difficulty washing hands often (AOR: 1.263). Those living with HIV (AOR: 0.483) had significantly lower odds of a difficulty obtaining face mask.

## Discussion

The study findings showed that West Africans who reported depression faced significant challenge in obtaining face masks and in washing hands often. Nevertheless, they had no significant difficulty in working remotely. Their adherence to social distancing did not differ significantly from the rest of the population. Those living with HIV were less likely to have difficulty washing their hands often and did not differ significantly from the rest of the population with respect to keeping other COVID-19 prevention methods. Respondents with high risk for COVID-19 had significantly higher odds of social distancing and difficulty obtaining face masks than the rest of the population. Also, while respondents who had a close friend who tested positive for SARS-CoV-2 and who knew someone who died of COVID-19 had significantly higher odds of social distancing, those who tested positive for SARS-CoV-2 had significantly lower odds of social distancing and working remotely. In addition, those who had symptoms of COVID-19 but did not get tested had significantly lower odds of social distancing but significantly higher odds of working remotely. The findings only partially supported the study hypothesis and showed differences in using COVID-19 prevention measures by health and exposure to SARS-CoV-2 infection among residents in West Africa.

One of the main strengths of the present study is the novel insights gained from a region with scarce publication on the subject matter. The insights could inform the development of future action plans to mitigate the risk for COVID-19 for populations at high risk of contracting the infection, and other similar infections in the future. Also, the data collection instrument was available in multiple languages making it possible to include respondents from multiple cultures in the study.

The study is, however, a cross-sectional and cannot confirm causality. Also, although large, the sample is a convenience sample that is skewed largely those with tertiary education. This may have likely resulted from the unintended exclusion of persons who may not have access to the internet or who did not have a smartphone, as these are persons more likely to have lower education status and lower economic power. The data were collected during the first wave of the COVID-19 pandemic in the region and thus, data collection was restricted to online surveys since conventional surveys were not feasible [53]. Online surveys are subject to selection bias [54] and respondents may not be representative of general populations. Another study limitation was the data skewed towards respondents from Nigeria and Ghana, thus limiting the extent to which findings from this study can be extrapolated to countries in the subregion poorly represented or not represented in the data collected. In addition, data on time of HIV diagnosis was not collected and challenges related to false positive diagnoses have been noted during the pandemic [55]. Despite these limitations, the results provide suggestive information that can inform COVID-19 and other similar epidemic/pandemic mitigation plans for the region.

First, we observed that respondents with predisposing health risks to COVID-19 appear to understand their risk and therefore, are better at physical distancing than the rest of the population. Those with little or no health risk are more likely to engage in risk-taking [56]. However, they are less able to obtain face masks than the general population. The pandemic had driven up the demand and cost of face masks limiting access to them in sub-Saharan Africa [57]. The cost of face masks was identified as a factor limiting the use of face masks [58]; and it may be more challenging for persons with health problems who have additional out-of-pocket expenditure on health care [59]. One support measure may be the provision of free face masks to visitors of healthcare facilities and this can be offered by donors and philanthropists.

Men and younger people were more likely to have difficulty obtaining face masks. Young people may find it difficult to obtain a face mask due to cost. The findings seem to support our postulation as those with reduced wages had higher odds of reporting difficulty obtaining a face mask. This, however, does not explain why males would not be able to obtain as face mask as males are more economically buoyant than non-males in the region [60]. However, males are less likely to perceive health risks [61], and pay attention to their health needs which may explain their higher risk for COVID-19 even in the region [62, 63].

Second, respondents living with HIV did not differ significantly from the rest of the population with respect to using COVID-19 prevention measures except that they seemed to have less difficulty washing their hands often. Less than 50% of the residents of West Africa has access to handwashing facilities at home [64]. However, people living with HIV may have received more education for hand hygiene practices [65] that have made them better prepared for the pandemic. They are, however, not better than the rest of the public with respect to other COVID-19 prevention measures. This singular practice may have helped reduce the risk of people living with HIV contracting COVID-19 as observed in Nigeria [66]. The water, sanitation and hygiene (WASH) programme taking place in the region needs to include COVID-19 prevention as a core element of its activities.

Third, we observed a paradox: people who had a close friend who tested positive SARS-CoV-2 and who knew someone who died of COVID-19 had significantly higher odds of social distancing while those who tested positive for SARS-CoV-2 had significantly lower odds of social distancing including working remotely. This may be connected with the perception of risk. Knowing someone who had COVID-19 may increase the perception of risk and adoption of precautionary measures. Conversely, a history of testing positive for SARS-CoV-2 may make individuals feel less concerned about personal risks because they think they are immune to further infection. The risk compensation theory suggests that people adjust their behaviour in response to perceived levels of risk and become more careful where they sense greater risk [67–71]. A few studies found no evidence of risk compensation during the pandemic [56, 68, 72], but our findings suggest it exists like it does in Bangladesh [56]. This finding may have implications for health education on the use of COVID-19 control measures. The public needs to appreciate that the prevention measures work for self and for others also. The concept of caring for others should not be strange for residents of West Africa who live in communitarian societies where the emphasis is on the connection between the individual and community members [73–75].

The present study also observed that individuals with symptoms of COVID-19 who did not get tested had significantly lower odds of physical distancing and significantly higher odds of working remotely. This may reflect high risk-taking nature of individuals who had symptoms of COVID-19 but did not get tested which would explain the poor physical distancing practices. Risk-takers have lower COVID-19 precautionary index [56]. However, it does not explain the higher odds of working remotely. Further studies are needed to explain these findings.

Finally, we observed that reported depression was associated with higher odds of inability to obtain face mask and difficulty washing hands often but higher odds of working remotely. Working remotely is associated with increased mental health challenges, including depression [76]. Individuals who are depressed may feel comfortable with being socially isolated [77, 78] and thereby, find working remotely a viable COVID-19 prevention strategy. This, however, is linked to health problems like sleep deprivation, impaired executive function and immunity, poor cardiovascular function and accelerated cognitive decline [79]. The difficulty to obtain face mask and wash hands often during the pandemic may also be connected with the reduced volition associated with depression [80]. Persons who report depression should be supported to access face masks and wash their hands often; and their indulgence with self-isolation should be addressed.

## Conclusions

Overall, this study signifies a disparity in the access to and use of COVID-19 preventive measures that is aligned with the health and COVID-19 status of residents in West Africa. Present findings point to risk compensation behaviours and this needs to be explored further.

## Supplementary Information

**Additional file 1.** List of countries in West Africa number of respondents included in the analysis.

## Data Availability

The datasets generated and/or analysed during the current study are not publicly available due to ongoing analysis using the dataset but are available from the corresponding author on reasonable request.

## Abbreviations

AOR: Adjusted Odds Ratio
COVID-19: Corona Virus Disease 2019
HIV: Human Immunodeficiency Virus
SARS-CoV-2: Severe acute respiratory syndrome coronavirus 2
WASH: Water, Sanitation and Hygiene
