# Supplementary material for: Is self-reported depression, HIV status, COVID-19 health risk profile and SARS-CoV-2 exposure associated with difficulty in adhering to COVID-19 prevention measures among residents in West Africa?
Source: BMC Public Health. 2022 Nov 10;22:2057. doi: 10.1186/s12889-022-14429-6 (PMC9648438; doi:10.1186/s12889-022-14429-6)
Supplement: Supplementary file 1 — Additional file 1. List of countries in West Africa number of respondents included in the analysis. [file 12889_2022_14429_MOESM1_ESM.docx]

Supplementary file 1: List of countries in West Africa number of respondents included in the analysis

| S/no | Country | Number of respondents | Percentage of sample |
| --- | --- | --- | --- |
| 1 | Benin | 12 | 0.2 |
| 2 | Burkina Faso | 3 | 0.1 |
| 3 | Cote d'Ivoire | 32 | 0.6 |
| 4 | Gambia | 9 | 0.2 |
| 5 | Ghana | 363 | 7.2 |
| 6 | Liberia | 18 | 0.4 |
| 7 | Mali | 31 | 0.6 |
| 8 | Mauritania | 1 | 0.0 |
| 9 | Nigeria | 4563 | 90.4 |
| 10 | Senegal | 10 | 0.2 |
| 11 | Sierra Leone | 7 | 0.1 |
| 12 | Togo | 1 | 0.0 |
|  | Total | 5050 | 100.0 |
